# AAV1.tMCK.NT-3 gene therapy improves phenotype in *Sh3tc2^−/−^* mouse model of Charcot–Marie–Tooth Type 4C

**DOI:** 10.1093/braincomms/fcae394

**Published:** 2024-11-06

**Authors:** Burcak Ozes, Lingying Tong, Kyle Moss, Morgan Myers, Lilye Morrison, Zayed Attia, Zarife Sahenk

**Affiliations:** Center for Gene Therapy, The Abigail Wexner Research Institute, Nationwide Children’s Hospital, Columbus, OH 43205, USA; Center for Gene Therapy, The Abigail Wexner Research Institute, Nationwide Children’s Hospital, Columbus, OH 43205, USA; Center for Gene Therapy, The Abigail Wexner Research Institute, Nationwide Children’s Hospital, Columbus, OH 43205, USA; Center for Gene Therapy, The Abigail Wexner Research Institute, Nationwide Children’s Hospital, Columbus, OH 43205, USA; Center for Gene Therapy, The Abigail Wexner Research Institute, Nationwide Children’s Hospital, Columbus, OH 43205, USA; Center for Gene Therapy, The Abigail Wexner Research Institute, Nationwide Children’s Hospital, Columbus, OH 43205, USA; Center for Gene Therapy, The Abigail Wexner Research Institute, Nationwide Children’s Hospital, Columbus, OH 43205, USA; Department of Pediatrics and Neurology, Nationwide Children’s Hospital, The Ohio State University, Columbus, OH 43210, USA; Department of Pathology and Laboratory Medicine, Nationwide Children’s Hospital, Columbus, OH 43205, USA

**Keywords:** NT-3, CMT4C, Sh3tc2, AAV

## Abstract

Charcot–Marie–Tooth Type 4C (CMT4C) is associated with mutations in the SH3 domain and tetratricopeptide repeats 2 (*SH3TC2*) gene, primarily expressed in Schwann cells (SCs). Neurotrophin-3 (NT-3) is an important autocrine factor for SC survival and differentiation, and it stimulates neurite outgrowth and myelination. In this study, scAAV1.tMCK.NT-3 was delivered intramuscularly to 4-week-old *Sh3tc2^−/−^* mice, a model for CMT4C, and treatment efficacy was assessed at 6-month post-gene delivery. Efficient transgene production was verified with the detection of NT-3 in serum from the treated cohort. NT-3 gene therapy improved functional and electrophysiological outcomes including rotarod, grip strength and nerve conduction velocity. Qualitative and quantitative histopathological studies showed that hypomyelination of peripheral nerves and denervated status of neuromuscular junctions at lumbrical muscles were also improved in the NT-3-treated mice. Morphometric analysis in mid-sciatic and tibial nerves showed treatment-induced distally prominent regenerative activity in the nerve and an increase in the estimated SC density. This indicates that SC proliferation and differentiation, including the promyelination stage, are normal in the *Sh3tc2^−/−^* mice, consistent with the previous findings that Sh3tc2 is not involved in the early stages of myelination. Moreover, in size distribution histograms, the number of myelinated axons within the 3- to 6-µm diameter range increased, suggesting that treatment resulted in continuous radial growth of regenerating axons over time. In conclusion, this study demonstrates the efficacy of AAV1.NT-3 gene therapy in the *Sh3tc2^−/−^* mouse model of CMT4C, the most common recessively inherited demyelinating CMT subtype.

## Teaser

Ozes *et al*. report that adeno-associated virus serotype 1 neurotrophin-3 gene therapy improved functional and electrophysiological outcomes in a mouse model of Charcot–Marie–Tooth disease Type 4C. The treatment mitigated hypomyelination state and increased myelinated fibre density distally and neuromuscular junction connectivity in peripheral nerves. Reduced muscle fibre size was also improved.

## Graphical Abstract

**Graphical Abstract:**
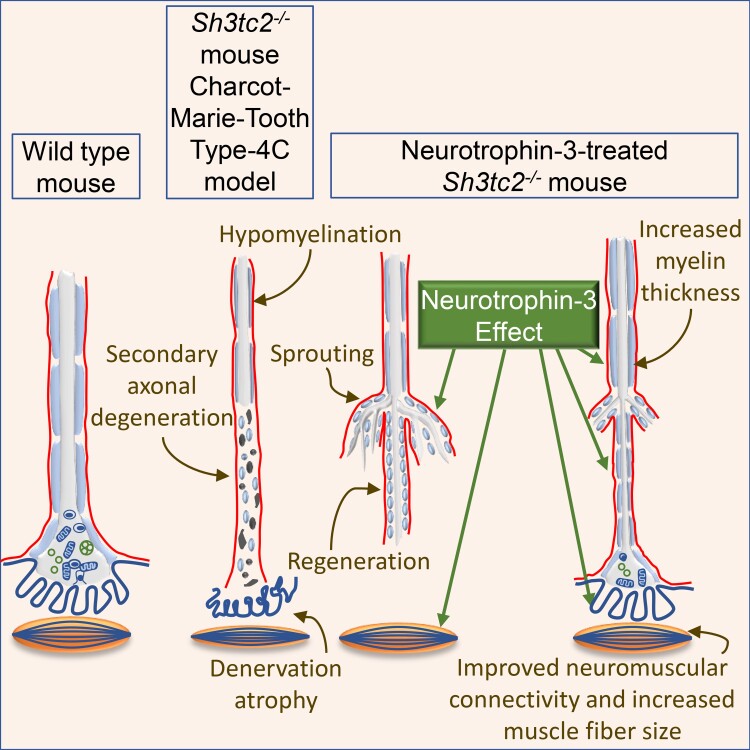

## Introduction

Charcot–Marie–Tooth (CMT) disease is a clinically and genetically diverse group of inherited peripheral neuropathies. CMT Type 4C (CMT4C) is an autosomal recessive form of the CMT subtype due to bi-allelic variants in the SH3 domain and tetratricopeptide repeats 2 (*SH3TC2*) gene.^1^ CMT4C typically is classified as a demyelinating type, characterized by early onset spinal deformities, and occasional cranial nerve deficits associated with variable moderate-to-severe disability.^2-4^ The *Sh3tc2^−/−^* mouse model of CMT4C adequately recapitulates the clinical phenotype of progressive peripheral neuropathy, manifested by decreased motor and sensory nerve conduction velocity (NCV) and hypomyelination, starting at 4 weeks of age.^5^ The *SH3TC2*-encoded protein is specifically expressed in Schwann cells (SCs) and localizes to the plasma membrane and several components of perinuclear endocytic recycling compartments that affect receptor dynamics, required for proper myelination and the integrity of the node of Ranvier.^5-7^  *In vitro* studies have shown that this localization is altered in CMT4C mutants leading to loss of intracellular targeting of SH3TC2, loss of Rab11 binding and loss of function on the endocytic recycling pathway.^6-8^

The *Sh3tc2^−/−^* mouse model was recently used to assess the potential for gene replacement therapy in CMT4C using a SC-specific promoter and lentivirus vector via intrathecal delivery, resulting in effective localization of SH3TC2 to the SC cell cytoplasm, with partial improvement of motor function and motor conduction velocities.^9^ Although this and a similar study in which adeno-associated virus (AVV) serotype 9 was used^10^ has served as proof of principal for gene replacement therapy in CMT4C, the translational potential of an intrathecal route that could lead to efficient number of SC transduction in human peripheral nerves remains questionable. This scepticism is simply based on the obvious gross and microscopic anatomical differences between mouse and human sciatic nerves.^11-14^ Therefore, it is unlikely that a mechanism of proximo-distal virus spread through diffusion, as proposed in the mouse sciatic nerve, could directly be applicable to the human counterpart. The feasibility of SC transduction via intrathecal delivery should be thoroughly tested in non-human primates prior to giving serious consideration for the potential use of this approach in clinical trials aimed correction of primary SC genetic defects.

We have long been aware of the daunting obstacles in developing gene therapy approaches for CMT subtypes with primary SC genetic defects. In newborn pups, while endothelial tight junctions are still open, a dose-dependent green flourescent protein expression in SCs can easily be achieved following systemic delivery of AAV vectors. Contrary, in addition to the blood–nerve barrier, the unique haemodynamics of circulation and gross and microanatomical characteristics of peripheral nerves would likely to limit the access of viral vectors to SCs following systemic delivery in adult mice.^15-19^ This unmet challenge calls attention to the neurotrophin-3 (NT-3) gene therapy approach in which the transgene is directed towards correcting impaired pathophysiology in peripheral nerves. NT-3, a versatile peptide of small molecular weight, is an important autocrine factor for SC survival and differentiation and stimulates neurite outgrowth and myelination. In prior studies, the ability of mutant SCs to respond to exogenously delivered NT-3 in peptide form by a subcutaneous route was demonstrated in two different experimental paradigms and in a small cohort of patients with myelin protein 22 duplication of CMT1A. These proof of principle studies showed that NT-3 significantly improved axonal regeneration and enhanced the myelination process in experimental models and in sural nerves from patients with CMT1A, improving neuropathy impairment score and reducing sensory deficit as well.^20^ However, a short serum half-life, high production costs, need for repeated dosing and discontinuation of the commercial availability of product were limitations that discouraged further clinical trials of subcutaneously injected NT-3. A more advantageous delivery system for NT-3 was developed using self-complimentary (sc) AAV serotype 1 as a vehicle. The scAAV1.tMCK.NT-3 construct expresses human NT-3 cDNA under the control of skeletal muscle-specific tMCK promoter. This so-called surrogate gene therapy approach entails intramuscular (IM) delivery of the scAAV1.tMCK.NT-3 vector, providing a systemic effect following transduction of muscle to produce NT-3 protein, which is released into serum continuously, as detected by ELISA (enzyme-linked immunosorbent assay).^21-26^

In the current study, we demonstrate the efficacy of AAV1.NT-3 gene therapy in the *Sh3tc2^−/−^* mouse model of CMT4C leading to significant functional, electrophysiological and histological improvements. In view of the current potential obstacles in reaching SCs for targeted gene therapy, and the fact that the majority of CMT subtypes are due to mutations in myelin genes, we believe that NT-3 gene therapy is well positioned with potential for providing efficacy in this group of CMT patients.

## Materials and methods

### Animals and treatment groups

*Sh3tc2^−/−^* mouse model, also known as *Sh3tc2^ΔEx1^*, (JAX stock #033933) was previously generated and described.^5^ All animal experiments were performed according to the guidelines approved by The Research Institute at Nationwide Children’s Hospital Animal Care and Use Committee that operates full accordance with the Animal Welfare Act and the Health Research Extension Act (Institutional Animal Care and Use Committee approval number: AR18-00076). PCR analysis was performed for genotyping. Fifteen (seven females and eight males) *Sh3tc2^−/−^* mice received 1 × 10^11^ vg dose of rAAV1.tMCK.NT-3 vector via IM injection into the gastrocnemius muscle at 1 month of age. Age- and sex-matched *Sh3tc2^−/−^* mice injected with Ringer’s lactate were included in the study as controls (*n* = 16, eight females and eight males). Outcome measures included functional assays involving rotarod and grip strength tests, and electrophysiology, which were performed in a blinded fashion, and detailed quantitative histopathological studies of the peripheral nervous system and muscle, and analysis of innervation status of neuromuscular junctions (NMJs). The mice were sacrificed 6 months following NT-3 gene treatment, at 7 months of age. At the end-point, mice were euthanized and blood, sciatic and tibial nerves, and selected hind limb muscles were collected.

### rAAV1.tMCK.NT-3 vector production and potency

Self-complementary recombinant AVV serotype 1 vector expressing the human NT-3 transgene under the control of muscle-specific tMCK promoter was designed as described previously.^21^ Viral Vector Core at Nationwide Children’s Hospital, Columbus produced the vector. Blood samples were collected from anaesthetized mice at end-point and vector potency was tested using ELISA as described before to measure serum NT-3 levels.^21^

### Functional outcomes

*Sh3tc2^−/−^* mice were tested using rotarod and grip strength at baseline (1 day before injection, 1 month of age), mid-point (3-month post-injection, 4 months of age) and end-point (6-month post-injection, 7 months of age). Nerve conduction studies were also performed at baseline and end-point.

#### Rotarod

Accelerating rotarod instrument (Columbus Instruments, OH, USA) was used to test the motor function and balance of *Sh3tc2^−/−^* mice via an. Acclimation run was performed at least 24 h before the experimental run. The protocol was run at 5 rpm with a constant acceleration of 0.5 rpm/s, and the best run out of the three runs was included in the analysis.

#### Grip strength

Bilateral simultaneous hind limb grip power was obtained using a grip strength meter (Chatillon Digital Meter, Model DFIS-2, Columbus Instruments, Columbus, OH, USA). Animal grasping the platform of the instrument was pulled back until it released the platform, and this step was repeated three times per mouse. Each force measurement was documented, and average of these three measurements was included in the analysis.

#### Nerve conduction studies

Two per cent isoflurane was used to anaesthetize the animals and mice were kept on a heating pad during measurements to maintain body temperature. Nicolet Viasys Viking Select EMG EP System (Nicolet Biomedical, WI, USA) and 27 G disposable needle electrodes were used on the right sciatic to measure NCV as described previously.^24^

### Nerve histology

The sciatic and tibial nerves were removed under a dissecting microscope and were fixed in glutaraldehyde and processed for plastic embedding using already established routine protocols.^27^ One-micrometer-thick, toluidine blue stained cross-sections from mid-sciatic and distal half of tibial nerves were used for quantitative analyses. Samples were excluded from analysis only if they were not suitable based on criteria including staining quality, contrast and artefacts such as wrinkles in the section. Outcomes of behavioural or physiological analyses are not considered in the exclusion/inclusion process.

#### G ratio and myelinated fibre size distribution analysis in sciatic nerves

G ratio is used to assess myelin thickness and is calculated by dividing axonal diameter with fibre diameter. Two random non-overlapping areas of sciatic sections were photographed for each mouse at ×90 magnification (Nikon Eclipse Ti2-E, Japan). NIS-Elements software was used to develop a recipe for an automated, unbiased g ratio calculation. Software utilizes the colour contrast between axon and myelin to mark them and automatically calculates g ratio of each fibre. An average of 1699.3 ± 78.2 and 1592.2 ± 76.4 fibres per mouse were analysed for treated (*n* = 8, equal sex distribution) and untreated mice (*n* = 9; four females and five males), respectively, to calculate g ratio, generate per cent g ratio distribution histograms and myelinated fibre size distribution histograms.

#### Abnormal SC density analysis in sciatic nerves

Data from each mouse were derived from randomly selected five non-overlapping areas, photographed at ×100 magnification using an Olympus BX41 microscope and SPOT Insight 12Mp sCMOS camera. A number of SCs with abnormally distended cytoplasm containing osmiophilic bodies were determined from each mouse from untreated, AAV1.NT-3-treated *Sh3tc2^−/−^* mice and wild-type (WT) controls (*n* = 10 for each cohort of *Sh3tc2^−/−^* mice; *n* = 5 for WT; equal sex distribution in each cohort). The mean number of abnormal SCs is expressed per 0.1 mm^2^ of endoneurial area.

#### Ultra-structural morphometric analysis for mitochondria density determination

Ultra-structural morphometric studies were performed using cross-sectional images of sciatic nerves at ×10 000 final magnification. Twenty-three randomly selected myelinated fibres with axon diameters around 3–5 µm selected from *Sh3tc2^−/−^* and WT mice. For each fibre, the profiles of axonal mitochondria and axonal cross-sectional areas were determined, and the mean mitochondrion number was expressed per 10 µm^2^ unit of axon.

#### Myelinated fibre size distribution and estimated SC density analysis in tibial nerves

Data from each mouse were derived from randomly selected areas, photographed at ×100 magnification using an Olympus BX41 microscope and SPOT Insight 12Mp sCMOS camera.

Axon diameter measurements were obtained from the computer screen image frames using BioQuant TCW14 Life Sciences imaging software (BioQuant Image Analysis Corporation; Nashville, TN, USA). The mean total measurements per mouse from the tibial nerves was 470.9 ± 17.2 in the treated and 388.9 ± 19.6 in the untreated cohorts (*n* = 7 for each cohort). Composites of fibre size distribution histograms and the mean myelinated fibre densities [mean ± standard error of the mean (SEM), number/0.1 mm^2^] were generated. For estimated SC density analysis, images from 11 treated (*n* = 5 females and *n* = 6 males) and nine untreated mice (*n* = 5 females and *n* = 4 males) were used for SC nuclei profiling. The sum of the number of SC nuclei associated with unmyelinated axon–SC complexes and those associated with unmyelinated axons at promyelination stage as well as the number of myelinated fibres constituted the estimated total SC density for each mouse.^20^

### Immunohistochemical analysis of NMJs

A previously published protocol was used.^28^ Shortly, lumbrical muscles were fixed in 4% paraformaldehyde and incubated in blocking buffer (5% horse serum, 5% bovine serum albumin and 1% Triton X-100 in phosphate buffered saline). After incubation in primary antibodies (acetylcholine receptor antibody, α-bungarotoxin, T1175, 1:500; anti-neurofilament 200 antibody, N4142, 1:500; SV2 antibody, AB_ 2315387, 1:50 described previously),^29,30^ tissues were incubated in secondary antibodies (Alexa Fluor 488-conjugated anti-rabbit and anti-mouse IgG, 1:500). NMJs were photographed using Nikon AX R confocal microscope. 127.3 NMJs were counted per mouse in average (*n* = 4 for treated and *n* = 3 for untreated). Innervation status of the NMJs was interpreted as published previously.^31^ Status of NMJs was determined as innervated when nerve and acetylcholine receptors were well co-localized, as partially innervated when co-localization is partial and as denervated when there is no co-localization.

### Muscle histology

Gastrocnemius and tibialis anterior muscles were collected and 12-μm-thick cross-cryostat sections were subjected to succinic dehydrogenase enzyme histochemistry to assess metabolic fibre-type differentiation using the standard protocol established in our laboratory.^32^ One representative image from deep, intermediate and superficial zones of the muscle sections was photographed at ×20 magnification using an Olympus BX41 microscope and SPOT Insight 12 Mp sCMOS camera. Staining quality, contrast and lack of artefacts were considered during selecting samples; functional or physiological outcomes were not regarded. Fibre types were determined based on staining intensity: slow-twitch oxidative fibres were most densely stained, fast-twitch oxidative fibres were intermediately stained, and fast-twitch glycolytic fibres were lightly stained. The shortest distance across the muscle fibre was measured as fibre diameter (Zeiss Axiovision LE4 software V4.9.1.0), and mean fibre diameter (mean ± SEM) was calculated for each fibre type (slow-twitch oxidative, fast-twitch oxidative and fast-twitch glycolytic) as well as for combination of all fibre types. Data were obtained from a total of 5404 and 4828 fibres of the treated cohort (*n* = 10, each cohort) and 6121 and 5914 fibres of the untreated cohort (*n* = 11, each cohort) for gastrocnemius and tibialis anterior, respectively.

### Statistical analysis

GraphPad Prism 9.0 software was used for all statistical analyses. Two-tail Student *t*-test, one-way ANOVA with Tukey’s multiple comparison test, two-way ANOVA with Sidak’s multiple comparison test and linear regression analysis were performed when applicable, and the significance level was set at *P* ≤ 0.05. The tests that meet the best assumptions of the data were chosen. Data were reported mean ± SEM in all experiments. *n* numbers, name of the statistical analysis and explanation of each data point shown in the plots were given in figure legends. Sample size for each experiment was established based on our previous studies with analogous experiments.^24,25^ Blinding was not used except the functional tests. Since recognizable treatment effects were present, blinding was not feasible for histopathological analysis.

## Results

### rAAV.NT-3 vector production and potency

Design and production of scAAV1.tMCK.NT-3 (Supplementary Fig. 1A) were performed at Nationwide Children`s Hospital, Columbus as described previously.^21^ scAAV1.tMCK.NT-3 was injected to the gastrocnemius muscle of *Sh3tc2^−/−^* mice at 4 weeks of age. Blood samples were collected from treated and untreated mice at 6-month post-gene delivery, and serum NT-3 levels were determined by a capture ELISA as previously reported (Supplementary Fig. 1B).^21^

### Efficacy of NT-3 gene therapy in *Sh3tc2^−/−^* mice

#### Functional and electrophysiological studies

**Figure 1:**
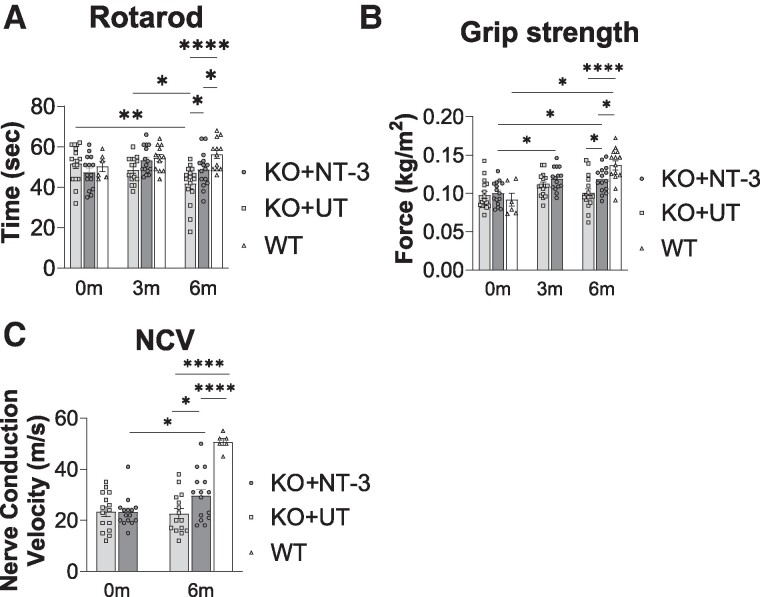
**NT-3 gene therapy improves functional outcomes in *Sh3tc2^−/−^* mice.** (**A**) Bar graphs show rotarod data at baseline (0 months: 0 m), at 3-month post-gene delivery (3 m) and end-point, which is 6-month post-gene delivery (6 m), (6 m, NT-3: 48.67 ± 2.26 s, *n* = 15 versus UT: 41.69 ± 2.45 s, *n* = 16, *P* = 0.0381; UT, 0 m: 51.63 ± 2.25 s versus 6 m: 41.69 ± 2.45 s, *n* = 16 each, *P* = 0.0014). Each data point represents the best rotarod run out of the three runs for each mouse. (**B**) Bar graphs show grip strength data at 0, 3 and 6 m (6 m, NT-3: 0.1182 ± 0.004 kg/m^2^, *n* = 15 versus UT: 0.1006 ± 0.005 kg/m^2^, *n* = 16, *P* = 0.0297; NT-3, 0 m: 0.1002 ± 0.004 kg/m^2^ versus 6 m: 0.1182 ± 0.004 kg/m^2^, *n* = 15 each, *P* = 0.0281). Each data point represents the average of three grip strength measurements for each mouse. (**C**) Bar graphs show NCV data at 0 and 6 m (6 m, NT-3: 29.73 ± 2.47 m/s, *n* = 15 versus UT: 22.67 ± 2.01 m/s, *n* = 16, *P* = 0.0256; NT-3, 0 m: 23.30 ± 1.52 versus EP: 29.73 ± 2.47, *n* = 15 each, *P* = 0.0480). Each data point represents one NCV measurement for each mouse. Data are represented as mean ± SEM; two-way ANOVA, Tukey’s multiple comparisons test; *P < 0.05, **P < 0.01, ***P < 0.001 and ****P < 0.0001.

Rotarod performance, tested at baseline, 3- (mid-point) and 6-month (end-point) post-treatment showed that NT-3 gene therapy in *Sh3tc2^−/−^* (also referred as KO in figures) mice preserved function well (47.33 ± 2.09 s at baseline, 53.20 ± 1.74 s at mid-point and 48.67 ± 2.26 s at end-point; *n* = 15). Comparatively, in the untreated *Sh3tc2^−/−^* controls (51.63 ± 2.25 s at baseline versus 41.69 ± 2.45 s at end-point; *n* = 16, *P* = 0.0014), there was a steady decline, despite a higher mean performance level at baseline than the treated group (Fig. 1A). At end-point, rotarod performance of the untreated mice dropped significantly, by 26%, compared with WT (WT, 56.33 ± 2.17 s, *n* = 12 versus UT, 41.69 ± 2.45 s, *n* = 16; *P* < 0.0001), while NT-3 treatment compensated this drop by half, to 13.6%; the end-point performance of the NT-3-treated mice was significantly better than the UT counterparts (NT-3: 48.67 ± 2.26 s, *n* = 15 versus UT: 41.69 ± 2.45 s, *n* = 16; *P* = 0.0381). Females performed better than males at all time points for all cohorts, which was statistically significant only in the untreated cohorts (Supplementary Fig. 2A); there was no significant sex-specific response to NT-3 gene therapy.

Grip strength of treated and untreated cohorts was also tested at baseline, mid-point and end-point. Compared with baseline, treatment improved grip strength at the mid-point testing, which continued to remain significant at 6-month post-treatment (end-point), while no significant change was observed in the untreated cohort during this time period (Fig. 1B). Consequently, the end-point grip strength performance following NT-3 gene transfer in the *Sh3tc2^−/−^* mice was significantly better than the untreated counterparts (NT-3, 0.118 ± 0.004 kg/m^2^, *n* = 15 versus UT, 0.101 ± 0.005 kg/m^2^, *n* = 16; *P* = 0.0297). Males performed better than females at all time points in all cohorts, although the difference was significant only in the untreated cohorts (Supplementary Fig. 2B). Therefore, we did not observe a sex-specific response to treatment in this test as well.

Sciatic nerve conduction outcome studies supported the efficacy of AAV1.NT-3 gene therapy (Fig. 1C). The mean NCV in the untreated *Sh3tc2^−/−^* mice at end-point did not change from baseline, which was about 44.8% of the WT (UT: 22.67 ± 2.01 m/s, *n* = 16, versus WT: 50.67 ± 1.36 m/s, *n* = 6, *P* < 0.0001). We observed further slowing of the NCV in the untreated *Sh3tc2^−/−^* mice at 10 months (15.00 ± 1.08 m/s; *n* = 4), compatible with the progression of the neuropathic process with aging. The end-point NCVs were significantly higher in the treated cohort (NT-3: 29.73 ± 2.47 versus UT: 22.67 ± 2.01 m/s; *n* = 15 in each cohort, *P* = 0.0256), which corresponded to a 31.2% improvement. Higher, but not statistically significant, NCVs were obtained from females at both time points, although no sex-specific response to treatment was observed (Supplementary Fig. 2C).

#### Morphological studies

**Figure 2:**
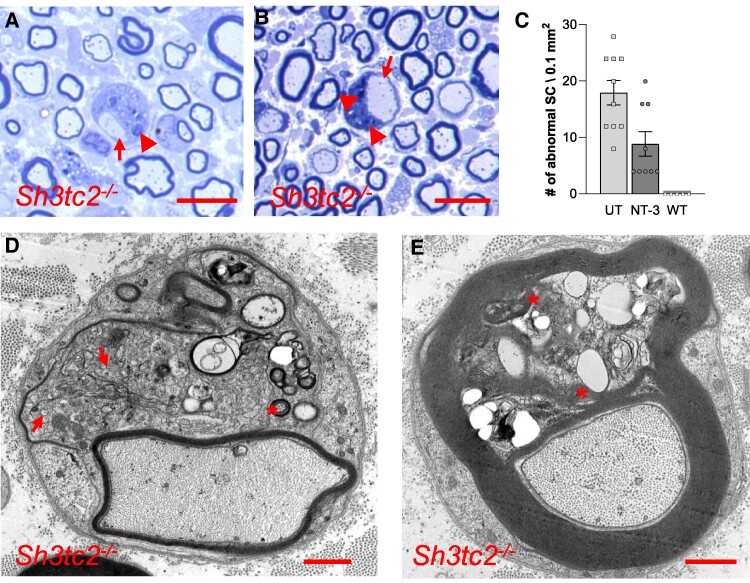
**Schwannopathy in *Sh3tc2^−/−^* mice.** (**A**, **B**) Toluidine blue stained semi-thin plastic sections from sciatic nerves showing thinly myelinated and solitary naked axons (arrows) and osmiophilic inclusions within distended SC cytoplasm (arrow heads). (**C**) The quantification of abnormal SCs in the untreated (UT) and AA1.NT-3 treated (NT-3) nerves showing a significant decrease in their number per a unit cross-sectional area of endoneurium with treatment; these abnormal SC profiles were not seen in WT (UT: 17.93 ± 2.2, *n* = 10 versus NT-3: 8.85 ± 2.2, *n* = 9, *P* = 0.0090; WT = 0, *n* = 5). Each data point represents number of abnormal SCs\0.1 mm^2^ calculated based on the abnormal SCs counted on randomly selected five non-overlapping areas per mouse. (**D**, **E**) Electron microscopic images from sciatic nerve of a Sh3tc2*^−/−^* mouse showing SC cytoplasm, distended with accumulations of tubular and vesicular membranous organelles of various sizes (arrows) and myelin figures (asterisk). Scale bar for **A** and **B**: 10 µm; for **D** and **E**: 1 µm. Data are represented as mean ± SEM; unpaired *t*-test; **P < 0.01.

Our phenotypic characterization studies in sciatic nerve samples from *Sh3tc2^−/−^* mice showed the presence of hypomyelination state, at 1 month of age, which continued to progress with aging as described previously.^5^ Thinly myelinated and solitary naked axons were frequently encountered in samples from the untreated cohort at 7 months of age (Fig. 2A–C). Rare profiles of Wallerian degeneration and onion bulb formations (as evidence of demyelination/remyelination) were identified, although not prominent as reported previously.^5^ We also noted the presence of osmiophilic inclusions within the myelinating and promyelinating SC cytoplasm, increased with aging (Fig. 2A and B). These abnormal SCs were decreased in the treated nerves and not present in age-matched WT controls (Fig. 2C). At the ultra-structural level, these inclusions corresponded to compacted membrane profiles with uncompacted loose extensions (Fig. 2D and E). In addition, SC cytoplasm was distended with accumulations of tubular and vesicular membranous organelles of various sizes (Fig. 2D and E). These membranous profiles and tubulovesicular structures were noted frequently in adaxonal, abaxonal and incisural (Schmidt–Lanterman) cytoplasmic compartments, compatible with a manifestation of impaired membrane recycling. In addition, unmyelinated axon–SC complexes appeared simplified with a notable axon loss, and SC processes engulfing empty collagen packets giving the appearance of ‘abnormally branched processes of non-myelinating SCs’ as previously described in CMT4C patients (Supplementary Fig. 3A). Within these axon–SC complexes, the presence of rudimentary axons surrounded by several layers of SC processes without compaction was common. A significant finding also not emphasized previously was the presence of an obvious increase of neurofilament content with increased packing density seen in numerous thinly myelinated axons compared with comparable size WT axons (Supplementary Fig. 3B–D). These alterations in the neurofilament content and spacing might be considered as a secondary axonopathy that resulted from Schwannopathy due *Sh3tc2^−/−^* status of SCs, and impaired SC–axon interactions.^33,34^ Moreover, these axons commonly displayed excessive number of mitochondria supported by morphometric analysis (Supplementary Fig. 3E) suggesting stagnation, an impairment in their transport.^33,34^

**Figure 3:**
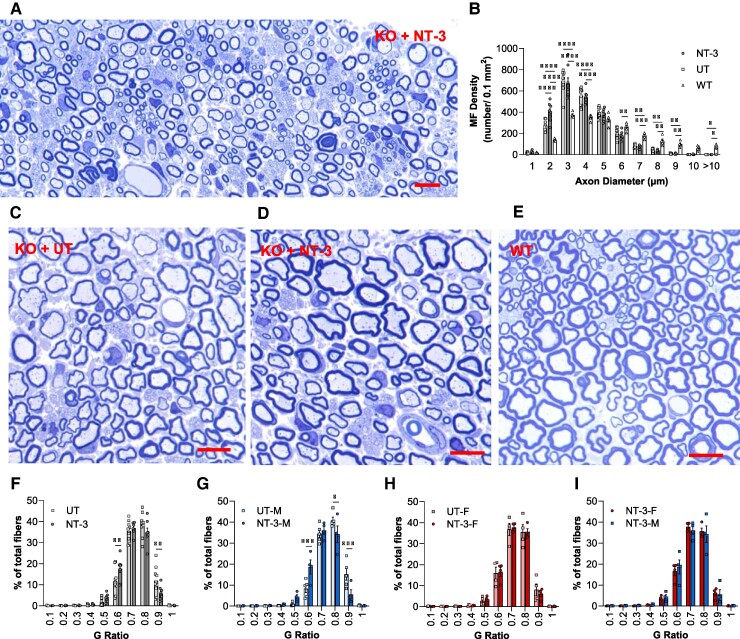
**Histological improvements in sciatic nerve of *Sh3tc2^−/−^* mice at 6-month post-NT-3 gene transfer.** (**A**) Representative semi-thin, toluidine blue–stained cross-section of sciatic nerve from NT-3 treated mice. **(B)** Myelinated fibre density distribution shows an increase in the small myelinated fibres with diameter ≤ 4 µm (NT-3, *n* = 8; UT, *n* = 9; WT, *n* = 5). Each data point represents mean myelinated fibre/0.1 mm^2^ per mouse. Semi-thin sciatic nerve section from (**C**) untreated, (**D**) NT-3 treated and (**E**) WT mice. (**F**) G ratios shown as percentage distribution indicate a shift towards smaller G ratios with NT-3 gene transfer therapy in *Sh3tc2^−/−^* mice (both sexes combined). (**G**, **H**) Shift is prominent in the treated males having more severe hypomyelination phenotype than females. (**I**) Treated cohorts do not show a sex-dependent difference in the g ratio percentage distribution. (**F–I**) Each data point represents percentage g ratio distribution per mouse. Scale bar: 10 µm. Data are represented as mean ± SEM; two-way ANOVA, Tukey’s multiple comparisons test; *P < 0.05, **P < 0.01, ***P < 0.001 and ****P < 0.0001. NT-3-F, treated females; NT-3-M, treated males; UT-F: untreated females; UT-M, untreated males.

At 6-month post-gene transfer, we noted an increase in the number of small myelinated axons in some specimens (Fig. 3A). As illustrated in the myelinated fibre size distribution histograms at the mid-sciatic level, *Sh3tc2* mutants displayed a significantly greater number of small-diameter myelinated fibres (with axons <4-µm diameter) compared with age-matched WT, with a paucity of the large diameter fibre population and increased myelinated fibre density (number per unit area) along with a smaller endoneurial cross-sectional area of sciatic nerve (Fig. 3B). We found that the treatment-induced increase in the number of small myelinated fibres was more prominent in females (Supplementary Fig. 4), although the mean myelinated fibre density was not altered with treatment (NT-3: 2287.7 ± 113.7/0.1 mm^2^ versus UT: 2259.4 ± 72.8/0.1 mm^2^, *P* = 0.8328; both sexes combined). There was, however, a visible increase in the myelin thickness in the treated cohort compared with the untreated counterparts, closer to WT nerves (Fig. 3C–E), supported by the g ratio (axon diameter/fibre diameter) reduction, corroborating the electrophysiological and functional studies. The average g ratio in the untreated *Sh3tc2^−/−^* mice was 0.70 ± 0.010 (*n* = 8, equal sex distribution), significantly greater than that obtained from our historical WT data (0.66 ± 0.002), reflecting the presence of thinner myelin in this model (Supplementary Table 1). Untreated males displayed thinner myelin than females (0.71 ± 0.009 versus 0.68 ± 0.015, *P* = 0.068). The AAV1.tMCK.NT-3 gene therapy led to a significant reduction in the mean g ratio (NT-3: 0.67 ± 0.008, *n* = 8, versus UT: 0.70 ± 0.010, *n* = 9, equal sex distribution; *P* < 0.0492) with gender influence in the *Sh3tc2^−/−^* mice (Supplementary Table 1) along with a reduction in the percentage of thinly myelinated fibres compared with the untreated cohort. The fibres with g ratio ≥ 0.7 constituted ∼77.7% of total myelinated fibres in the treated group (both sexes combined) while the same fibre population in the untreated cohort was ∼86.1% (Fig. 3F). Untreated males appeared more hypomyelinated than females; the percentage of fibres with g ratio ≥ 0.7 was 90.6% in males, while the same population was found lower, ∼80.4% in females (Fig. 3G and H). Although the effect of NT-3 appeared more prominent in males, and despite that they were more hypomyelinated to begin with, the percentage distribution of fibres with the same g ratio was identical in both sexes compatible with our previous observations that NT-3 exerts a normalization effect towards WT levels without sex difference (Fig. 3I).

**Figure 4:**
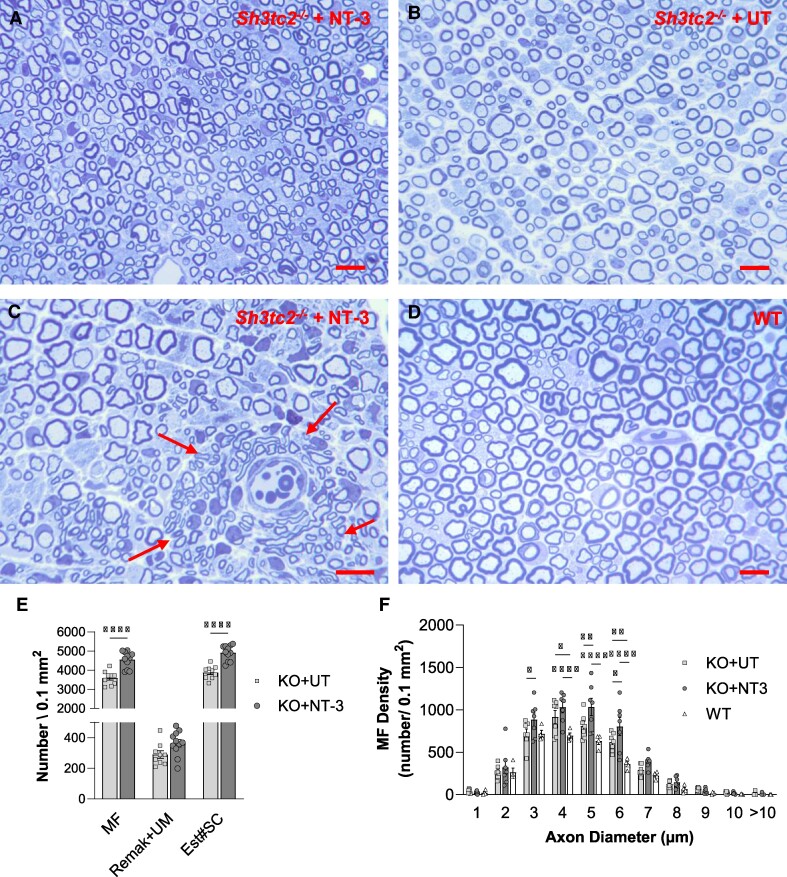
**Myelinated fibre increase in tibial nerve of *Sh3tc2^−/−^* mice following NT-3 gene transfer.** (**A**, **C**) Representative semi-thin, toluidine blue–stained cross-sections from tibial nerve of (**A**) treated and (**B**) untreated mouse and (**C**) arrows showing clustering of regenerating small myelinated fibres around an endoneurial blood vessel from a treated mouse. (**D**) WT tibial nerve at same magnification with **A** and **B** is enclosed for comparison. (**E**) Bar graphs show increases in the densities of myelinated fibre, the SC nuclei associated with Remak bundles and those associated with unmyelinated axons at promyelinating stage (Remak + UM) and the sum of these items as estimated SC (Est#SC) number per unite area in the treated *Sh3tc2^−/−^* mice compared with untreated counterparts (NT-3, *n* = 11; UT, *n* = 9). Each data point represents mean data point per mouse separately for myelinated fibre, Remak + UM and Est#SC (please see Materials and methods). (**F**) Myelinated fibre density distribution histograms show an increase in the number of myelinated axons for the subpopulation with axon diameter between 3- and 6-µm range with treatment (*n* = 7 for NT-3 and UT cohorts, *n* = 5 for WT cohort). Each data point represents mean myelinated fibre/0.1 mm^2^ per mouse. Scale bar: 10 µm. Data are represented as mean ± SEM; two-way ANOVA, Sidak’s multiple comparisons test; *P < 0.05, **P < 0.01, ***P < 0.001 and ****P < 0.0001.

Contrasting with the mid-sciatic nerve segments, quantitative studies from the tibial nerves revealed treatment-related increase in the mean myelinated fibre density (4688.6 ± 171.3/0.1 mm^2^ versus 3872.1 ± 195.0/0.1 mm^2^, *n* = 7 per cohort, both sexes combined; *P* = 0.0085) with an apparent increase in the small myelinated fibres compared with untreated *sh3ct2*^−/−^ nerves (Fig. 4A and B). Clustering of regenerating sprouts with a preferential perivascular localization was noted in three out of 11 mice (Fig. 4C), which were absent in the untreated (Fig. 4B) and WT tibial nerves (Fig. 4D). In addition to increased myelinated fibre density, we also found an increase in number of SC nuclei associated with unmyelinated axon–SC complexes (unmyelinated axons ensheathed by Remak SCs) and unmyelinated axons at promyelination stage, therefore resulting in a significant increase in the estimated SC number per unit area, calculated as the sum of these three entities (NT-3: 4917.3 ± 119.4/0.1 mm^2^, *n* = 11, versus UT: 3889.0 ± 114.7/0.1 mm^2^, *n* = 9; *P* < 0.0001; Fig. 4E). The myelinated fibre size distribution histograms showed that in the treated cohort, the number of myelinated fibres per unit area was significantly increased for a subpopulation of fibres with axon diameter between 3- and 6-µm range compared with the untreated, suggesting continuous radial growth of regenerating axons over time (Fig. 4F). In addition, contrasting with the previous mice models that we tested for the efficacy of AA1.NT-3 gene therapy, the circulating serum NT-3 levels in the *Sh3ct2*^−/−^ model were much higher, suggesting that *Sh3ct2* null Remak SCs were capable of proliferating, producing and releasing endogenous NT-3 upon stimulation by the exogenous, transgene-induced NT-3. The serum NT-3 levels at 6-month post-gene injection showed a correlation with the mean myelinated fibre densities, the estimated SC numbers in the nerves and the conduction velocities in the individual animals (Supplementary Fig. 5).

**Figure 5:**
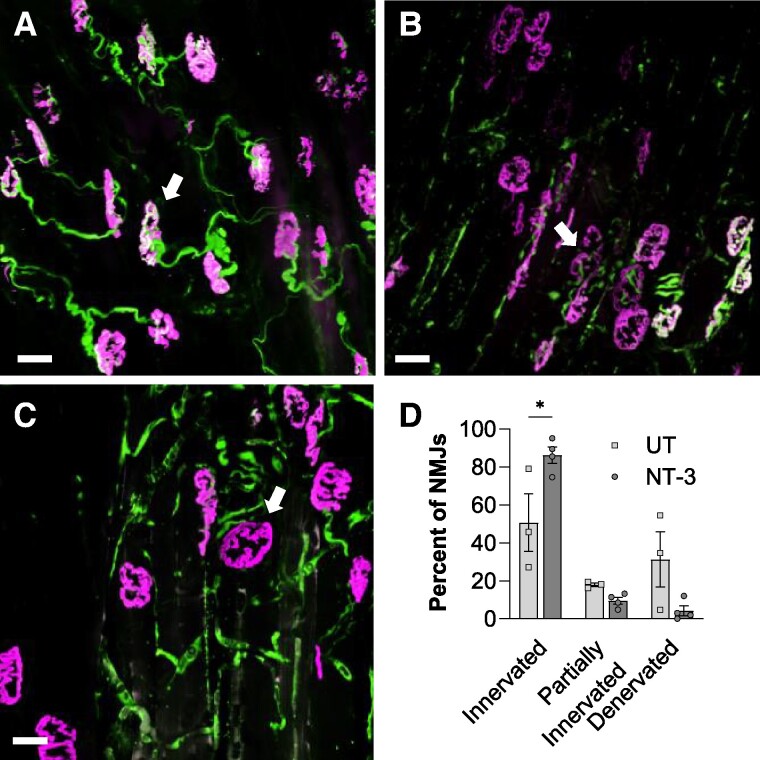
**NT-3 gene transfer improves NMJ integrity in *Sh3tc2^−/−^* mice.** (**A–C**) Representative images showing NMJs from the lumbrical muscles of the *Sh3tc2^−/−^* mice. Arrows indicate examples of **(A)** innervated, (**B**) partially innervated and (**C**) denervated NMJs (magenta: acetylcholine receptor antibody; green: anti-neurofilament 200 antibody and SV2 antibody; white: innervated areas). (**D**) Bar graph shows innervated NMJs are increased in the treated mice (NT-3: 86.24 ± 4.32%, *n* = 4, versus UT: 50.68 ± 15.15%, *n* = 3, *P* = 0.0146). Each data point represents percentage of NMJs calculated for each mouse. Data are represented as mean ± SEM; two-way ANOVA, Sidak’s multiple comparisons test, *P < 0.05. Scale bar: 25 µm.

Additional studies at 6-month post-gene delivery revealed the protective effects of NT-3 on the of NMJ integrity in an intrinsic foot muscle group, lumbricals. NMJ pathology was previously described in gastrocnemius muscle from 8-month-old *Sh3ct2*^−/−^ mice without changes in the innervating axon, suggesting that the compromised junctional integrity can be an early sign in CMT4C.^28^ Our quantitative analysis of NMJs in lumbricals revealed a significant increase in the percentage of innervated NMJs with treatment compared with the UT counterparts (NT-3:86.24 ± 4.32%, *n* = 4, versus UT: 50.68 ± 15.15%, *n* = 3; *P* = 0.0146; Fig. 5A–D).

We also analysed histopathological changes with AAV1.NT-3 gene therapy in anterior and posterior compartment muscles of distal leg in *Sh3tc2^−/−^* mice at 6-month post-treatment. Gastrocnemius and tibialis anterior muscles from untreated *Sh3tc2^−/−^* mice showed angular atrophic fibres, scattered or in small groups of belonging to either fast or slow twitch fibre subtypes, compatible with neurogenic changes (Supplementary Fig. 6A and B). AAV1.NT-3 treatment improved the mean fibre size in both muscles (Supplementary Tables 2 and 5) with a shift to larger diameter subgroups in size distribution histograms, and that this shift was prominent in the gastrocnemius muscle (Supplementary Fig. 6C and D). Compared with males, the treatment effects were observed in all fibre subtypes from females, being more prominent for Type 2 fibres, fast twitch oxidative and glycolytic subtypes (gastrocnemius, *P* = 0.045; tibialis anterior, *P* = 0.0199) (Supplementary Tables 3, 4, 6 and 7).

## Discussion

The findings we report here represent another example of the efficacy of AAV.NT-3 gene therapy in a mouse model for a CMT subtype that results from SC cell genetic defects. Mice with exon 1 of the *Sh3tc2* gene knocked out demonstrate many of the features seen in patients with CMT4C, which appears to be the most prevalent (18%) autosomal recessive CMT subtype.^1,2^ The clinical spectrum of CMT4C is broad, contrasting with the classical CMT phenotype having features of slowly progressive length-dependent distally prominent and symmetrical neuropathy. Common features of CMT4C include early onset of thoracic spine scoliosis, moderate-to-severe neuropathy with demyelinating features on NCV studies and cranial nerve deficits, suggesting the presence of several different pathobiological vulnerability sites, which may contribute to phenotypic variability. Increased intra- and interfamilial phenotypic variability in CMT4C has previously been attributed to the presence of cryptogenic modifiers or to the diversity of SH3TC2 protein function.^5,7,8,35-37^ Our own observations in the *Sh3tc2^−/−^* mouse model also suggest the presence of several vulnerability sites for potentially different pathomechanisms involved in CMT4C. These include Schwannopathy that primarily affects myelin production (hypomyelination state) and nodal alterations, Schwannopathy-induced secondary axonopathy (increased neurofilament density with presumed impairment of bidirectional organelle transport) and NMJ pathology. It is conceivable that early impairment of NMJ connectivity in the paraspinal muscles could account for the occurrence of childhood scoliosis or cranial nerve deficits in CMT4C, which are not commonly seen features of a classic CMT phenotype associated with a length-dependent distal axon loss. However, NMJ connectivity as early denervation sign has not been explored systematically, neither in this model nor in patients. In our study, as well as in the previous report,^28^ NMJ was studied later in the disease course. It should be noted that some CMT2D cases may present with early upper extremity involvement and develop severe scoliosis or cranial nerve deficits and that the mouse model for this CMT subtype also shows early NMJ defects,^25,31,38^ which may be responsible for those phenotypic manifestations that are not length dependent. Another vulnerability site noted in the *Sh3tc2^−/−^* mouse is the presence of secondary axonopathy manifested with abnormal neurofilament cytoskeleton along with increased mitochondria, suggesting impairment in mitochondrial transport, which we believe are likely factors contributing to length-dependent axonal loss seen in CMT4C patients.

Our study also provides evidence for morphologic correlates of the *Sh3tc2* null status of SCs in this model. Sh3tc2 is specifically expressed in SCs, localizing to the plasma membrane and perinuclear membranous structures of endocytic recycling compartment.^5,6^ It is reported that SH3TC2 acts as an effector of the small GTPase Rab11, a key regulator of recycling endosome functions.^36^ It has been suggested that *SH3TC2* mutations may lead to a disruption of this interaction resulting in myelination impairment and compromised SC elongation, leading to wider nodal gaps.^8,36^ It has also been proposed that certain specific mutations may likely cause hypomyelination by affecting the interaction between SH3TC2 and protein(s) other than Rab11,^3^ implying that functional domain-specific mutations may also be a factor in phenotypic variability. Nonetheless, we propose that the accumulation of compacted and non-compacted membranous profiles and tubulovesicular structures within SC cytoplasm in *Sh3tc2^−/−^* mice is likely the morphologic correlates of impaired membrane recycling/endosome functions that lead to dysfunctional SC, incapable of completing full myelination. SH3TC2 as an exclusive SC protein is expressed late in the myelination process, upregulated with myelination and not expressed in Remak cells.^37^ Our findings of increased SC number along with increased small myelinated fibres in response to NT-3 stimulation indicate that SC proliferation and differentiation, including the promyelinating stage, are normal in the *Sh3tc2^−/−^* mice, consistent with the previous findings that Sh3tc2 is not involved in the early stages of myelination.^37^ Moreover, we found several-fold higher serum NT-3 levels in the treated cohort compared with other CMT models that we studied. This suggests that the measured NT-3 levels may reflect a combination of exogenous NT-3, the transgene product and the endogenous NT-3. We propose that the circulating transgene product is stimulating endogenous NT-3 production by the *Sh3tc2^−/−^* Remak SCs, which are in a competent state for responding to NT-3 stimulation. SCs are known to express NT-3 and its preferred receptor TkrC, and NT-3 serves as an autocrine survival factor for SCs, stimulating SC survival and differentiation during embryonal development and in denervated SCs, in the absence of axonal contact.^39-42^

Contrasting with the mid-sciatic levels, we found significantly increased mean myelinated fibre density (number per unit area of 0.1 mm^2^) distally in tibial nerves from the treated *Sh3tc2^−/−^* mice compared with the untreated counterparts, compatible with distally prominent regenerative activity in the nerve. Although Wallerian degeneration profiles were few in each microscopic frame, it is likely that there is a compounding effect of axonopathy overtime distally in a length-dependent manner. In addition, the increase in the density of a subpopulation of fibres with axon diameters between 3 and 6 µm in the treated cohort, at 6-month post-treatment, indicates that there is a continuous radial growth of regenerating axons over time. In addition, the results also emphasize the presence of sex-dependent phenotypic differences in this model, males being more affected than females. The peripheral nerves from untreated males were more hypomyelinated, and, although not significant, males also had reduced number of myelinated fibres in the tibial nerves compared with their female counterparts. In addition, males underperformed females in baseline and end-point rotarod testing, although did better in grip strength without reaching statistical significance. NT-3 effect in both sexes, however, was equal, towards normalization indicating that, overall, males showed a greater percentage change when compared with baseline with treatment. As we previously reported, there can be potential sex-dependent phenotypic differences in disease models and the differences may need further considerations when assessing treatment efficacy.^24,25,32^

Previous proof of principle studies for gene replacement approach using a SC-specific promoter and viral vectors via intrathecal delivery into *Sh3tc2^−/−^* mice resulted in effective localization of Sh3tc2 to the SC cell cytoplasm, with partial improvement of motor function and motor conduction velocities.^9,10^ We are pleased to see that AAV1.NT-3 surrogate gene therapy approach in the same model produced comparable functional and electrophysiological improvements. In conclusion, we demonstrate the efficacy of AAV1.NT-3 gene therapy in the *Sh3tc2^−/−^*mouse model of CMT4C, the most prevalent of the recessively inherited demyelinating CMT subtype. Considering the fact that the majority of CMT subtypes are due to mutations in myelin genes, collectively, our studies indicate that NT-3 gene therapy is well positioned with the potential to provide disease-modifying effects for the largest CMT patient population.

## Supplementary Material

fcae394_Supplementary_Data

## Data Availability

The data that support the findings of the study will be made available from the corresponding author upon reasonable request.
