# Supplementary material for: AAV1.tMCK.NT-3 gene therapy improves phenotype in Sh3tc2−/− mouse model of Charcot–Marie–Tooth Type 4C
Source: Brain Commun. 2024 Nov 6;6(6):fcae394. doi: 10.1093/braincomms/fcae394 (PMC11562120; doi:10.1093/braincomms/fcae394)
Supplement: fcae394_Supplementary_Data [file fcae394_supplementary_data.pdf]

A

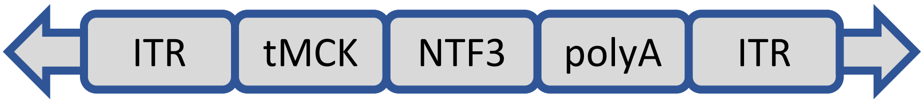

B

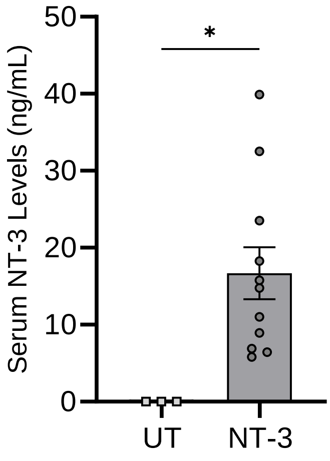

Supplementary Figure 1. scAAV1.tMCK.NT-3 vector diagram and serum NT-3 levels. (A) The diagram shows the NTF3 (gene bank designation of NT-3) is expression cassette composed of a tMCK (triple-tandem muscle creatine kinase) enhancer/promoter region (714 bp), the full-length NT-3 cDNA (774 bp) and the SV40 (simian virus 40) polyA tail (211 bp). Diagram is modified from Ozes et al., 2021[24] **(B)** Seven months old *Sh3tc2*$^{-/-}$ mice were injected with 1×10^{11} vg of scAAV1.tMCK.NT-3 vector, or with Ringer's lactate as control in the right gastrocnemius muscle. At endpoint (6 months post-gene delivery), serum samples were obtained from treated (NT-3) and untreated (UT) mice via cardiac puncture, and NT-3 levels were determined by ELISA. Each data represents ng/mL of NT-3 per animal. Error bars are ± SEM; *n* = 11 for treated cohort. NT-3 serum levels were with below detection range for UT mice (*n* = 3). Data is represented as mean ± SEM; unpaired t-test; p=0.0274.

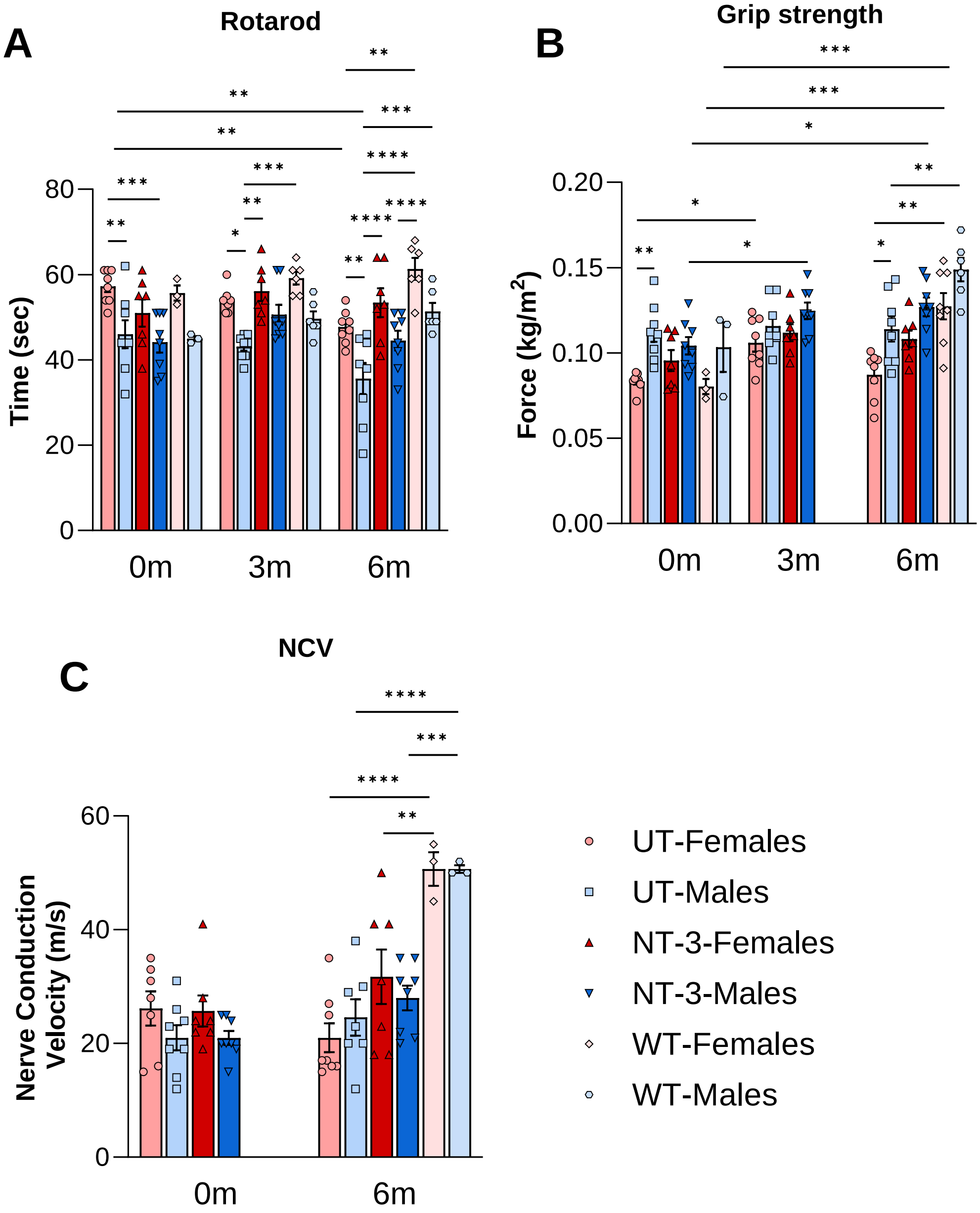

Supplementary Figure 2. Functional outcomes based on sexes. **(A)** Bar graphs show rotarod data at baseline (zero months: 0m), at three months post-gene delivery (3m) and endpoint, which is six-months post-gene delivery (6m) (0m, UT-Females: 57.25 vs UT-Males:46.0, n=8 for each, p=0.0063; 3m, UT-Females: 54.0 vs UT-Males: 43.13, n=8 for each, p=0.0093; 6m, UT-Females: 47.75 vs UT-Males: 35.63, n=8 for each, p=0.0024). Each data point represents the best rotarod run out of the three runs for each mouse. **(B)** Bar graphs show grip strength data at 0m, 3m and 6m (0m, UT-Females: 0.08335 vs UT-Males: 0.1123, n=8 for each, p=0.0082; 6m, UT-Females: 0.08725 vs UT-Males: 0.1140, n=8 for each, p=0.0187)). Each data point represents the average of three grip strength measurements for each mouse. **(C)** Nerve conduction velocity (NCV) data at 0m and 6m showed a trend higher for females but did not reach significance levels. Each data point represents one NCV measurement for each mouse. Legends at the part C applies to all. Data is represented as mean ± SEM; Two-way ANOVA, Tukey's multiple comparisons test; *$p < 0.05$, **$p < 0.01$, ***$p < 0.001$, ****$p < 0.0001$.

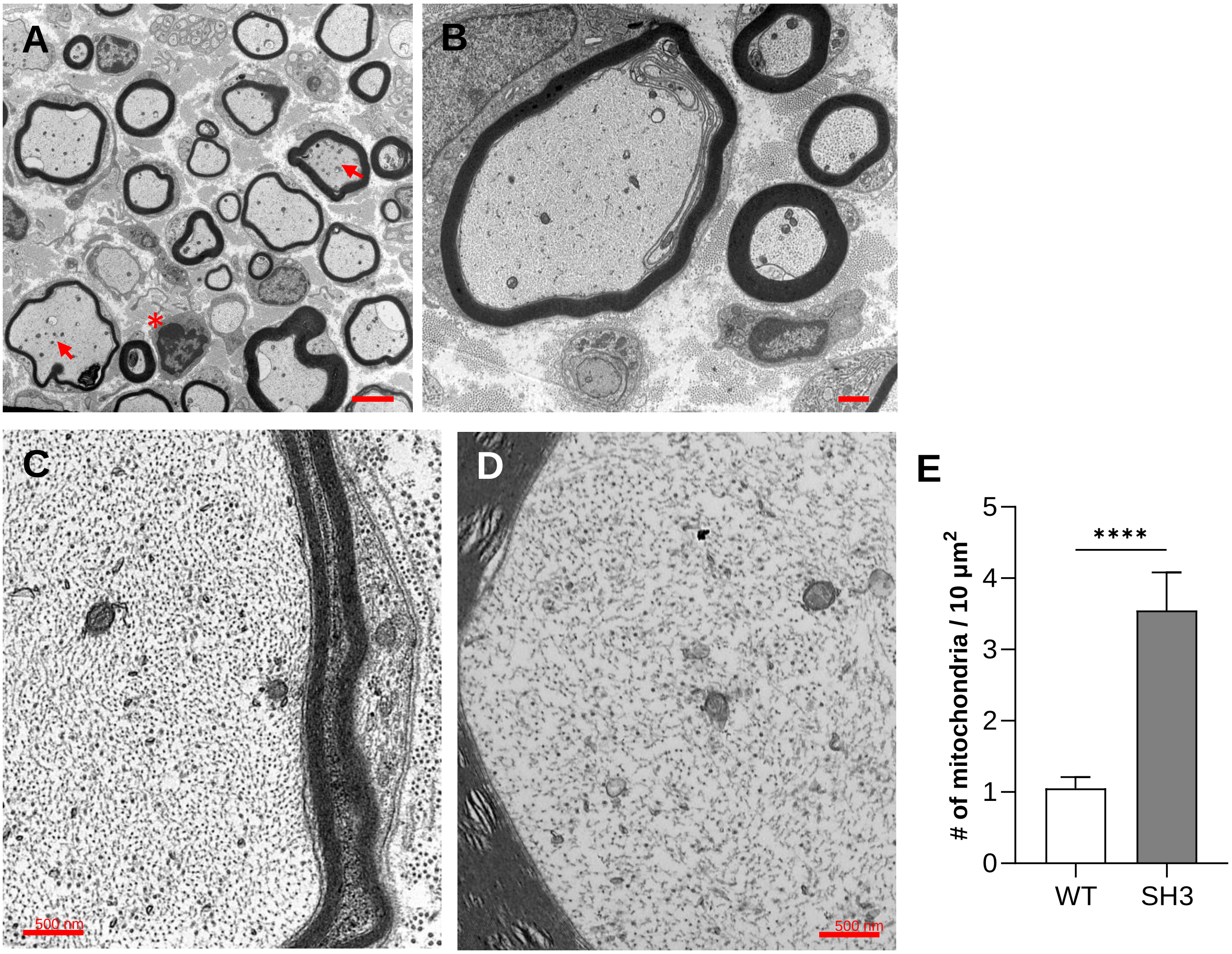

Supplementary Figure 3. Electron microscopy images from sciatic nerve of *Sh3tc2$^{-/-}$* mice. **(A)** Unmyelinated axon-Schwann cell (SC) complexes appear simplified with a notable axon loss, and SC processes engulfing empty collagen packets (asterisk). Excessive number of mitochondria (arrows) are present in some axons showing increased neurofilament content. **(B)** An obvious increase of neurofilament content with increased packing density **(C)** is seen in a thinly myelinated axon. **(D)** A comparable size WT axon shows normal neurofilament spacing for comparison in a WT mouse. **(E)** The number of mitochondria per unit area is increased in axons with increased neurofilament content from *Sh3tc2$^{-/-}$* mice compared to WT (n=23 comparable size axons per mouse from the sciatic nerve of one *Sh3tc2$^{-/-}$* mouse and WT mouse). Data is represented as mean ± SEM; unpaired t-test; ****p < 0.0001. Scale bars are 1 µm for (A, B) and 500 nm for (C, D).

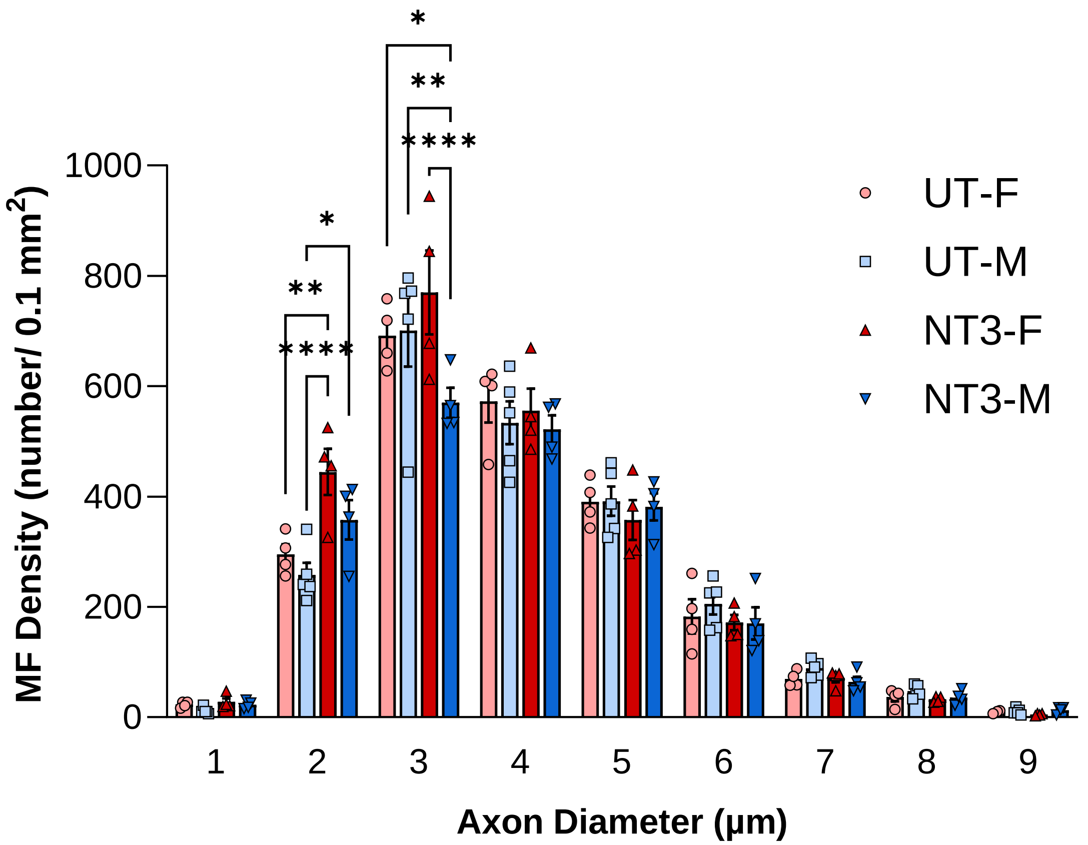

Supplementary Figure 4. Myelinated fiber (MF) density distribution based on sexes in sciatic nerve in *Sh3tc2*$^{-/-}$ mice. Myelinated fiber (MF) density distribution based on sexes in sciatic nerve shows that fiber size increase with diameter <3 µm observed in the treated cohort was more prominent in females. Each data point represents mean MF/0.1 mm2 per mouse. Data is represented as mean ± SEM; Two-way ANOVA, Tukey's multiple comparisons test; *p < 0.05, **p < 0.01, ***p < 0.001, ****p < 0.0001, n=4 for each group. UT-F: untreated females, UT-M: untreated males, NT3-F: NT-3 treated females, NT3-M: NT-3 treated males.

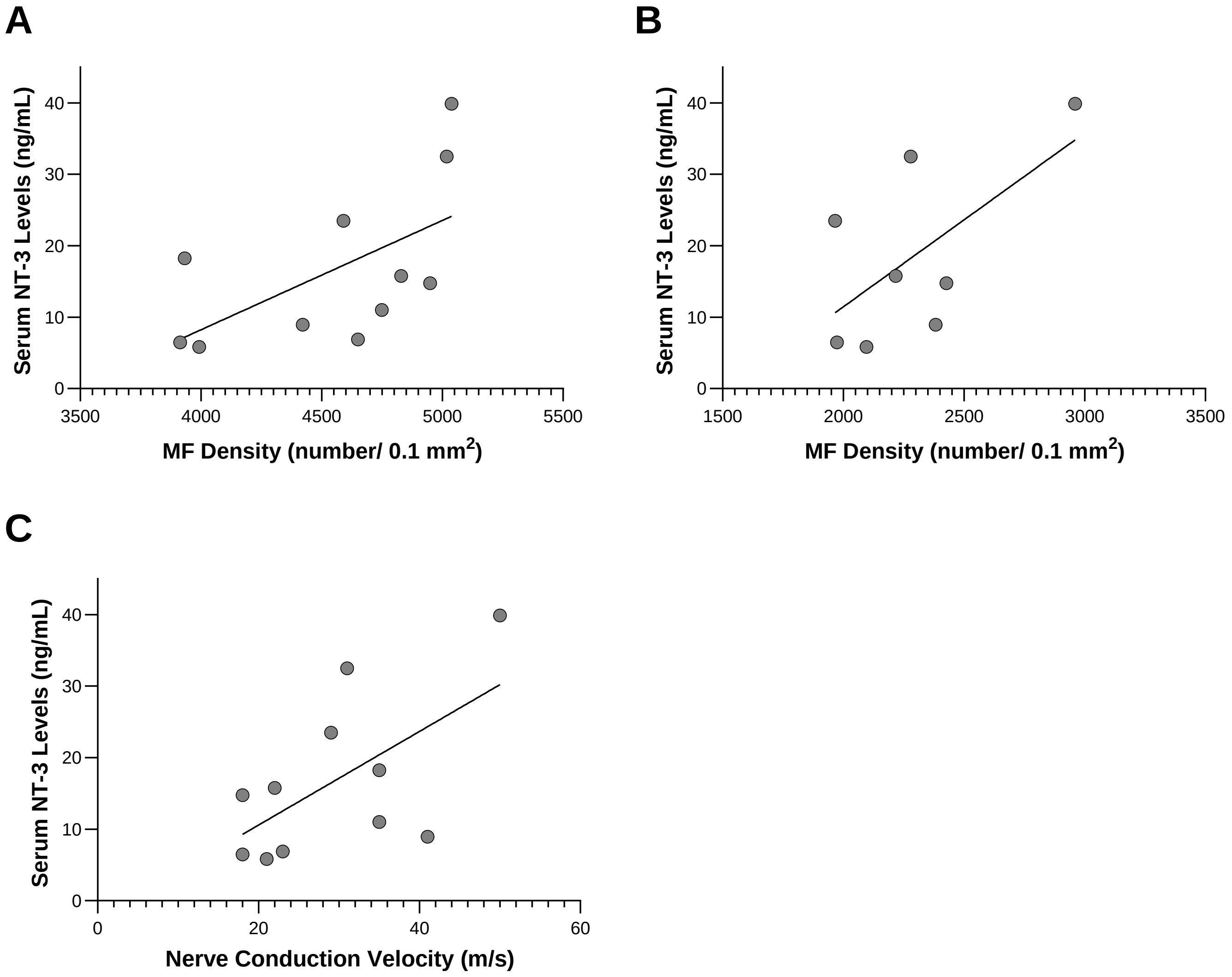

Supplementary Figure 5. Correlation of serum NT-3 levels with myelinated fiber (MF) density and nerve conduction velocity (NCV). Serum NT-3 levels show correlation with myelinated fiber (MF) density in **(A)** tibial (Simple linear regression, R^2=0.3478, n=11) and **(B)** sciatic nerves (Simple linear regression, R^2=0.3914, n=8) , and with **(C)** NCV values (Simple linear regression, R^2=0.3589. n=11). Paired data points for each mouse is shown.

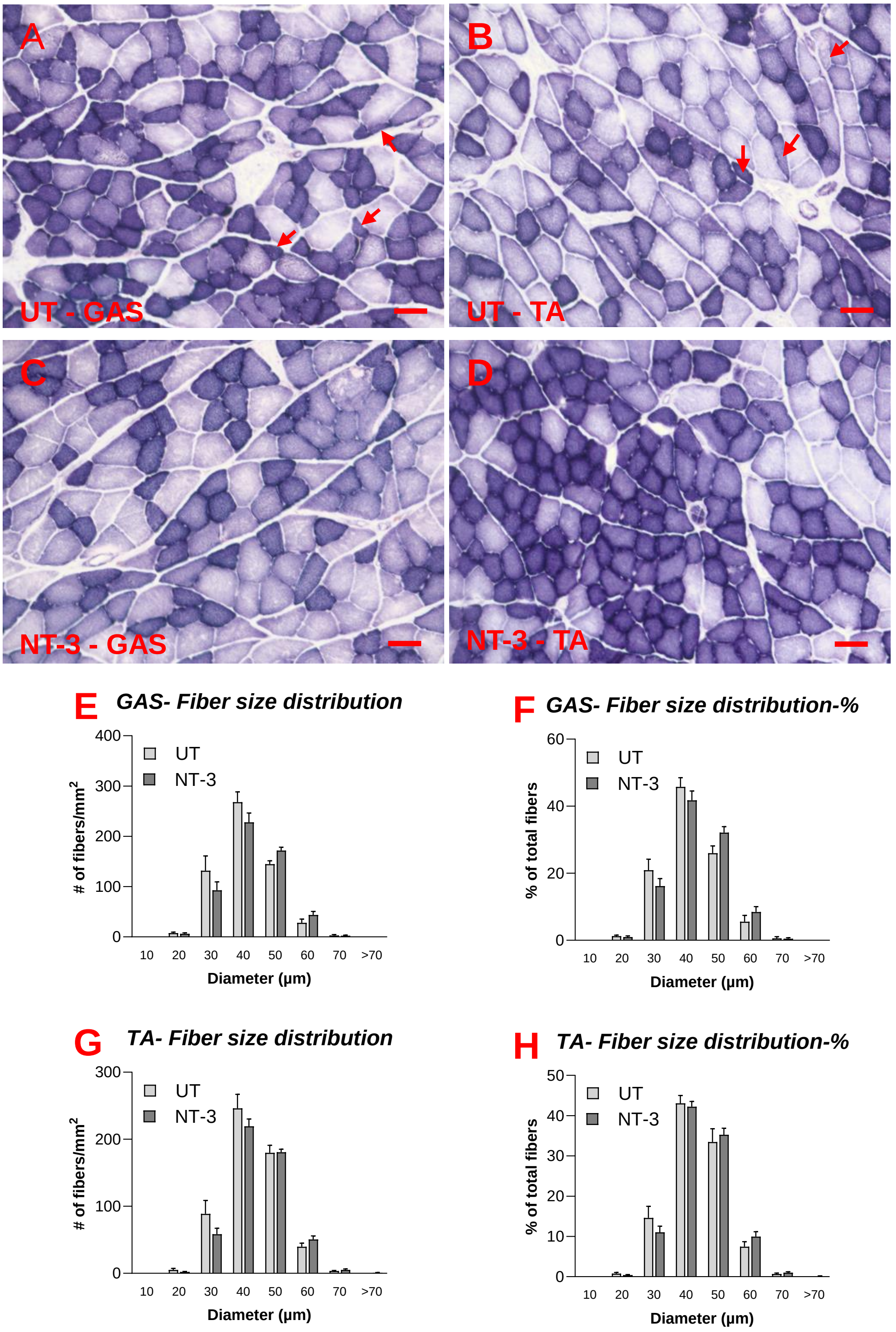

Supplementary Figure 6. Gastrocnemius and tibialis anterior muscle histology. Representative SDH images from **(A, B)** untreated and **(C, D)** treated **(A, C)** gastrocnemius (GAS) and **(B, D)** tibialis anterior (TA) muscles showing fiber size improvement in the treated muscles. Arrows mark the atrophic angular fibers in the untreated muscles. Muscle fiber size distribution graphs display a shift towards larger fibers in both **(E, F)** GAS (n=11 for UT, n=10 for NT-3) and **(G, H)** TA muscles (n=11 for UT, n=10 for NT-3) . Graphs represent **(E, G)** the fiber size density as well as **(F, H)** the percent distribution. Scale bar: 50 µm. Data is represented as mean ± SEM; Two-way ANOVA, Tukey's multiple comparisons test; *p < 0.05, **p < 0.01, ***p < 0.001, ****p < 0.0001.

Supplementary Table 1. Average G ratio of the treated and untreated *Sh3tc2*$^{-/-}$ mice.

Cohort	Average G ratio
NT-3 treated	0.67 ± 0.008*
Untreated	0.70 ± 0.010
NT-3 treated - Females	0.67 ± 0.007
Untreated - Females	0.68 ± 0.015
NT-3 treated - Males	0.66 ± 0.015*
Untreated - Males	0.71 ± 0.009

Data is represented as mean ± SEM, t-test between treated and untreated groups, *$p < 0.05$.

Supplementary Table 2. Fiber size analysis on gastrocnemius muscle of NT-3 treated and untreated *Sh3tc2*$^{-/-}$ mice.

	Untreated (n=11)		NT-3 Treated (n=10)	
	**Number**	**Diameter (µm)**	**Number**	**Diameter (µm)**
**STO**	228.8 ± 16.9	32.17 ± 1.0	242.6 ± 18.9	33.20 ± 0.8
**FTO**	230.5 ± 13.8	38.05 ± 0.9	189.8 ± 20.9	40.65 ± 1.3
**FTG**	97.2 ± 16.2	44.19 ± 1.2	108.0 ± 12.8	42.80 ± 1.5
**All Fibers**	556.5 ± 30.5	36.59 ± 1.0	540.4 ± 37.3	37.79 ± 1.0

Data represented as mean ± SEM, 2-way ANOVA, Tukey's multiple comparison test

Supplementary Table 3. Fiber size analysis on gastrocnemius muscle of NT-3 treated and untreated *Sh3tc2*$^{-/-}$ female mice.

	Untreated (n=5)		NT-3 Treated (n=4)	
	**Number**	**Diameter (µm)**	**Number**	**Diameter (µm)**
**STO**	221.8 ± 23.0	31.19 ± 1.5	250.8 ± 46.8	34.50 ± 0.9
**FTO**	222.2 ± 24.5	37.93 ± 1.5	179.0 ± 20.3	43.36 ± 1.6*
**FTG**	118.8 ± 27.0	42.72 ± 1.8	79.8 ± 22.3	44.91 ± 3.0
**All Fibers**	562.8 ± 49.5	36.05 ± 1.3	509.5 ± 51.9	39.66 ± 1.5

Data represented as mean ± SEM. *$p \leq 0.05$ (compared to UT), unpaired t-test.

Supplementary Table 4. Fiber size analysis on gastrocnemius muscle of NT-3 treated and untreated *Sh3tc2*$^{-/-}$ male mice.

	Untreated (n=6)		NT-3 Treated (n=6)	
	**Number**	**Diameter (µm)**	**Number**	**Diameter (µm)**
**STO**	234.7 ± 13.4	33.00 ± 1.4	237.2 ± 13.4	32.34 ± 1.2
**FTO**	237.3 ± 16.6	38.15 ± 1.2	197.0 ± 33.5	38.84 ± 1.6
**FTG**	79.2 ± 18.3	45.42 ± 1.7	126.8 ± 10.9	41.39 ± 1.5
**All Fibers**	551.2 ± 42.1	37.03 ± 1.5	561.0 ± 53.7	36.54 ± 1.1

Data represented as mean ± SEM, 2-way ANOVA, Tukey's multiple comparison test.

Supplementary Table 5. Fiber size analysis on tibialis anterior muscle of NT-3 treated and untreated *Sh3tc2*$^{-/-}$ mice.

	Untreated (n=11)		NT-3 Treated (n=10)	
	**Number**	**Diameter (μm)**	**Number**	**Diameter (μm)**
**STO**	257.3 ± 21.1	33.72 ± 0.9	232.8 ± 16.7	35.30 ± 0.8
**FTO**	183.2 ± 11.3	41.58 ± 0.9	171.3 ± 5.0	43.59 ± 0.8
**FTG**	97.2 ± 8.1	44.98 ± 0.8	78.7 ± 8.4	47.04 ± 1.0
**All Fibers**	537.6 ± 28.3	38.43 ± 0.9	482.8 ± 13.6	40.12 ± 0.6

Data represented as mean ± SEM, 2-way ANOVA, Tukey's multiple comparison test.

Supplementary Table 6. Fiber size analysis on tibialis anterior muscle of NT-3 treated and untreated *Sh3tc2*$^{-/-}$ female mice.

	Untreated (n=5)		NT-3 Treated (n=4)	
	**Number**	**Diameter (μm)**	**Number**	**Diameter (μm)**
**STO**	295.8 ± 25.7	32.40 ± 1.8	255.3 ± 15.5	35.52 ± 0.8
**FTO**	185.4 ± 22.1	40.99 ± 1.8	175.3 ± 5.7	43.00 ± 0.9
**FTG**	101.6 ± 14.1	44.49 ± 1.0	65.0 ± 14.5	48.70 ± 0.9*
**All Fibers**	582.8 ± 49.3	37.21 ± 1.8	495.5 ± 8.5	40.00 ± 0.5

Data represented as mean ± SEM. *p≤0.05 (compared to UT), unpaired t-test.

Supplementary Table 7. Fiber size analysis on tibialis anterior muscle of NT-3 treated and untreated *Sh3tc2*$^{-/-}$ male mice.

	Untreated (n=6)		NT-3 Treated (n=6)	
	**Number**	**Diameter (μm)**	**Number**	**Diameter (μm)**
**STO**	225.2 ± 27.3	34.83 ± 0.6	217.8 ± 25.6	35.16 ± 1.3
**FTO**	181.3 ± 12.0	42.07 ± 0.8	168.7 ± 7.7	43.99 ± 1.1
**FTG**	93.5 ± 10.3	45.38 ± 1.3	87.8 ± 9.0	45.93 ± 1.5
**All Fibers**	500.0 ± 26.4	39.44 ± 0.8	474.3 ± 22.1	40.20 ± 1.1

Data represented as mean ± SEM, 2-way ANOVA, Tukey's multiple comparison test.
